# A sialoadenectomy is associated with an increased risk of coronary heart disease: A three-year follow-up study

**DOI:** 10.1371/journal.pone.0199135

**Published:** 2018-06-18

**Authors:** Shih-Han Hung, Chin-Hui Su, Herng-Ching Lin, Chung-Chien Huang, Senyeong Kao

**Affiliations:** 1 Department of Otolaryngology, Taipei Medical University Hospital, Taipei, Taiwan; 2 Department of Otolaryngology, College of Medicine, School of Medicine, Taipei Medical University, Taipei, Taiwan; 3 Department of Otorhinolaryngology, Mackay Memorial Hospital, Taipei, Taiwan; 4 School of Health Care Administration, Taipei Medical University, Taipei, Taiwan; 5 School of Public Health, National Defense Medical Center, Taipei, Taiwan; Indiana University, UNITED STATES

## Abstract

Little is known regarding the long-term adverse effects of a sialoadenectomy. The purpose of this study was to estimate the risk of coronary heart disease (CHD) among patients receiving a sialoadenectomy procedure by utilizing a cohort study based on a population-based database in Taiwan. This study retrieved data of the study sample from the Longitudinal Health Insurance Database 2005. This retrospective cohort study included 608 patients who underwent a sialoadenectomy and 1824 propensity score-matched comparison patients. We individually tracked each sampled patient for a 3-year period from their index date to discriminate those who subsequently received a diagnosis of CHD during the follow-up period. We found that respective incidence rates of CHD during the 3-year follow-up period were 3.87 (95% confidence interval (CI): 3.01–4.91) and 1.79 (95% CI: 1.45–2.18) per 100 person-years for patients who did and those who did not undergo a sialoadenectomy. The stratified Cox proportional analysis revealed that the hazard ratio of CHD during the 3-year follow-up period was 2.43 (95% CI: 1.77–3.33) than comparison patients. This study demonstrates an association between sialoadenectomy and CHD.

## Introduction

Common salivary gland diseases generally originate from infections, sialolithiasis, congenital anomalies, and of course neoplastic diseases [1]. Sialoadenitis is often treated conservatively with antibiotics, salivary massage, and hydration [2]. Sometimes relieving the underlying obstruction is mandatory with the help of minimally invasive surgical procedures [3]. However, when the obstructive disease is too severe, or the salivary gland disorder is neoplastic in nature, a sialoadenectomy is traditionally recommended [4].

Little is known regarding the long-term adverse effects of a sialoadenectomy. Most studies focused on surgical complications with this procedure such as nerve damage and paralysis, remnant ductal tissue problems, or postoperative hemorrhage [5–7]. The risk of developing xerostomia under excessive gland removal was also mentioned [8]. Wase et al. first mentioned the possible effect of a sialoadenectomy on thyroid activity [9]. Navarivera et al. further reported the effect of a partial sialoadenectomy on thyroid gland function and structure, and proposed hormonal interrelationships of the salivary glands with other systems [10]. Removal of the submandibular gland was reported to be associated with lowered sperm production parameters in animals [11]. More recently, the importance of salivary-derived growth factors, including epidermal growth factor (EGF), that play a role in helping maintain levels of oral health by promoting wound healing and maintaining mucosal integrity, was addressed [12–14]. With such complex secretory components containing digestive enzymes, immunoglobulins, growth factors, electrolytes, and buffers, it seems that the actual long-term changes to the human body once the salivary glands have been removed remain largely unanswered [15, 16].

To date, increasing evidences indicated that salivary glands are important in nitrate transport [17]. In addition, the vasoprotective effects were considered to be associated with the activity of nitrite converted from ingested nitrate [18]. Thus, it was plausible that sialoadenectomy procedure might affect the enterosalivary conversion and further contribute to an elevated risk of CHD. The purpose of this study was to provide an estimation of risk of developing coronary heart disease (CHD) among patients receiving the sialoadenectomy procedure by utilizing a cohort study based on a population-based database in Taiwan.

## Methods

### Database

We retrieved data of the study sample from the Longitudinal Health Insurance Database 2005 (LHID2005). The LHID2005 consists of registration files and original medical claims for 1,000,000 randomly selected representative insurance enrollees listed in the 2005 Registry of Beneficiaries under the Taiwan National Health Insurance (NHI) program (*n* = 25.68 million). The LHID2005 allows researchers in Taiwan to longitudinally follow-up the utilization of medical services for these selected 1,000,000 enrollees.

This study was exempt from full review by the Institutional Review Board of National Defense Medical Center, since the LHID2005 consists of de-identified secondary data released to researchers for research purposes.

### Study sample

In this retrospective cohort study, we first identified 710 patients who underwent a sialoadenectomy (ICD-9-CM procedure code 26.3) between January 1, 2001 and December 31, 2010. We then excluded patients aged <18 years (*n* = 21) to limit the study sample to the adult population. We defined the date of the sialoadenectomy as the index date for these patients. We further excluded patients who had a history of CHD (ICD-9-CM codes 410~414 or 429.2) before their index date (*n* = 81). Ultimately, as a result, the study group included 608 patients who underwent a sialoadenectomy.

To select the comparison group, we first excluded all patients who had a history of a sialoadenectomy. However, since the Taiwan NHI began in 1995, the LHID2005 did not provide medical records before 1995. Consequently, we were unable to analyze the data prior to 1995 for all relevant analyses in this study. It was also impossible for us to exclude the possibility that some selected comparison patients might have undergone a sialoadenectomy before 1995. Nevertheless, this potential bias would lead the results toward the null. We randomly retrieved 1824 patients (three for every patient who underwent a sialoadenectomy) to match each patient who underwent a sialoadenectomy in terms of propensity score and the year of the index date using the SAS proc surveyselect program (SAS System for Windows, vers. 8.2, SAS Institute, Cary, NC). For the study group, the year of the index date was the year in which they underwent a sialoadenectomy. However, comparison patients were selected by matching them to a given patient who underwent a sialoadenectomy simply on their utilization of medical services in the same index year of that particular study patient. For the comparison group, we defined their first healthcare use occurring in the index year as their index date. Additionally, we have computed a propensity score for each patient. The patients’ demographics and comorbidities, including sex, age, urbanization level, monthly income, geographic region, hypertension, hyperlipidemia, diabetes, stroke, obesity, and tobacco use disorder were taken into a multivariable logistic regression model in order to calculate the possibility of receiving an sialoadenectomy. We also assured that none of the selected comparison patients had a history of CHD before their index date. We further assured that none of the comparison patients underwent a sialoadenectomy during the 3-year follow-up period.

As a result, 2432 patients were included in this study. We further individually tracked each sampled patient for a 3-year period from their index date to discriminate those who subsequently received a diagnosis of CHD during the follow-up period.

### Statistical analysis

All statistical analyses were performed with the SAS system (vers. 9.2; SAS Institute). We used Chi-squared tests to compare differences in sex, monthly income (NT$0~15,840, NT$15,841~25,000, ≥NT$25,001; the average exchange rate in 2013 was US$1.00≈New Taiwan (NT)$30), geographical location, urbanization level of the subject’s residence (five levels with 1 being the most urbanized and 5 being the least), and comorbidities between patients who underwent a sialoadenectomy and comparison patients. We further used stratified Cox proportional hazard regressions (stratified by propensity score and the year of the index date) to calculate the hazard ratio (HR) and its corresponding 95% confidence interval (CI) for the subsequent development of CHD during the 3-year follow-up period between patients who did and those who did not undergo a sialoadenectomy. We used a significance level of 0.05.

## Results

Of the 2432 total sampled patients in this cohort study, the mean age was 48.5±14.9 years (range 18–87 years). Table 1 shows the distributions of demographic characteristics and comorbidities stratified by the presence or absence of a sialoadenectomy. After being matched for propensity score, Table 1 reveals that there was no significant difference in sex (*p*>0.999), age (*p*>0.999), urbanization level (*p* = 0.443), monthly income (*p* = 0.274), and geographic region (*p* = 0.921) between patients who underwent a sialoadenectomy and comparison patients. As to comorbidities, we also failed to observe a significant difference in hypertension (*p* = 0.798), hyperlipidemia (*p*>0.999), diabetes (*p* = 0.395), stroke (*p*>0.999), obesity (*p* = 0.806), and tobacco use disorder (*p* = 0.215) between patients who did and those who did not undergo a sialoadenectomy.

**Table 1:** Demographic characteristics of sampled subjects (N = 2432).

Variable	Subjects who underwent a sialoadenectomy *N* = 608		Comparison subjects *N* = 1824		*p* value
	Total no.	Column %	Total no.	Column %	
Sex					
Male	350	57.6	1050	57.6	>0.999
Female	258	42.4	774	42.4	
Age (years)	48.5±14.9		48.5±14.9		>0.999
Urbanization level					0.443
1 (most)	189	31.1	523	28.7	
2	193	31.7	550	30.2	
3	89	14.6	312	17.1	
4	78	12.8	262	14.4	
5 (least)	59	9.7	177	9.7	
Monthly income					0.274
NT$0~15,840	203	33.4	675	37.0	
NT$15,841~25,000	241	39.6	683	37.5	
≥NT$25,001	164	27.0	466	25.6	
Geographic region					0.921
Northern	271	44.6	790	43.3	
Central	121	19.9	385	21.1	
Southern	203	33.4	609	33.4	
Eastern	13	2.1	40	2.2	
Hypertension	178	29.3	544	29.8	0.798
Hyperlipidemia	145	23.9	435	23.9	>0.999
Diabetes	98	16.1	268	14.7	0.395
Stroke	39	6.4	117	6.4	>0.999
Obesity	12	2.0	39	2.1	0.806
Tobacco use disorder	25	4.1	56	3.1	0.215

Note: The average exchange rate in 2008/2013 was US$1.00≈New Taiwan (NT)$29.

The incidence of CHD during the 3-year follow-up period is presented in Table 2. We found that respective incidence rates of CHD during the 3-year follow-up period were 3.87 (95% CI: 3.01–4.91) and 1.79 (95% CI: 1.45–2.18) per 100 person-years for patients who did and those who did not undergo a sialoadenectomy. The log-rank test suggests that patients who underwent a sialoadenectomy had a greater tendency to have CHD than comparison patients (*p*<0.001).

**Table 2:** Hazard ratio (HR) for coronary heart disease among sampled subjects during the 3-year follow-up period.

Presence of coronary heart disease	Total sample *(N* = 2432)		Subjects who underwent a sialoadenectomy (*N* = 608)		Comparison patients *(N* = 1824)	
	No.	%	No.	%	No.	%
3-year follow-up period						
Incidence rate per 100 person-years (95% CI)	2.30 (1.96–2.68)		3.87 (3.01–4.91)		1.79 (1.45–2.18)	
HR (95% CI)	-		2.43*** (1.77–3.33)		1.00	

Notes: CI, confidence interval. The HR was calculated by a stratified Cox proportional hazard regression which was stratified by propensity score and the year of the index date.

*** p<0.001

Furthermore, Table 2 shows the HR for CHD between patients who did and those who did not undergo a sialoadenectomy. The stratified Cox proportional analysis (stratified by propensity score and the year of the index date) revealed that the HR of CHD during the 3-year follow-up period was 2.43 (95% CI = 1.77–3.33) for patients who underwent a sialoadenectomy compared to comparison patients.

Table 3 analyzed the HRs of CHD between patients who did and those who did not undergo a sialoadenectomy according to sex. We found that both male and female patients who underwent a sialoadenectomy had a higher following risk of CHD than comparison patients (with an HR of 2.32 for males and 2.62 for females).

**Table 3:** Hazard ratios (HRs) for coronary heart disease among sampled subjects during the 3-year follow-up period by sex.

Presence of coronary heart disease	Sex							
	Males				Females			
	Subjects who underwent a sialoadenectomy (*N* = 350) *n*, %		Comparison patients (*N* = 1050) *n*, %		Subjects who underwent a sialoadenectomy (*N* = 258) *n*, %		Comparison patients (*N* = 774) *n*, %	
3-year follow-up period								
HR (95% CI)	2.32*** (1.55–3.46)		1.00		2.62*** (1.56–4.40)		1.00	

Notes: CI, confidence interval. The HR was calculated by a stratified Cox proportional hazard regression which was stratified by propensity score and the year of the index date.

*** p<0.001

## Discussion

In this study, we demonstrated that sialoadenectomy was significantly associated with subsequent incidence of CHD regardless of sex. Our findings support the salivary gland possibly having important roles that are interrelated with different systems across the human body, and the conventional thinking that the salivary glands are one of the relatively dispensable organs in the body should be re-evaluated.

The importance of the salivary glands and the extent of the sacrifice they can impart on the human body are truly debatable issues. Studies reporting various complications such as nerve damage and paralysis, remnant ductal tissue problems, and postoperative hemorrhage were limited in scope to immediate or short-term surgical procedure-related problems [5–7]. What about functional aspects? The most straightforward thinking about consequences after a sialoadenectomy is a reduction in the saliva amount. Dry mouth, xerostomia, and even oral candidiasis were largely addressed in various animal studies [19, 20]. However, little has been reported regarding these issues in humans; probably the problem largely depends on highly variable, age-dependent resting salivary flow, which outweighs the effect of a sialoadenectomy [21]. Similarly, little has been reported regarding the potentially decreased immunoglobulin amount in saliva after a sialoadenectomy and its consequences. What would be more interesting would be to examine those substances that were previously underlooked but were proven to have roles in normal physiology. Previously, we mentioned the reported impacts on thyroid activity and hormonal interrelationships after a sialoadenectomy [9, 10]. More recently, contributions of epidermal growth factor (EGF) by the salivary gland were emphasized. The importance of this growth factor was revealed through some animal studies decades ago [22–24]. This salivary gland-excreted growth factor is known to be associated with the development of gastric lesions [25]. Recently Ainola et al. reported that diminished salivary EGF secretion could be a link between Sjögren’s syndrome and autoimmune gastritis [26]. Azuma et al. also reported that the salivary EGF levels could play a role in the pathogenesis of refractory intraoral manifestations in Sjögren’s syndrome patients [27].

The sialochemistry behind the possible link between a sialoadenectomy and the development of CHD is likely to be mediated through nitrite metabolism. The importance of nitrite in the saliva has been known since 1974, when Tannenebaum et al. reported the possible relationship of salivary nitrite with nitrosamine formation [28]. Tenovuo et al. reported the biochemistry of nitrates, nitrites, and nitrosamines in human saliva [29]. Nitrate, after its absorption in the upper gastrointestinal tract, reaches the salivary glands via the blood circulation, where it is secreted into the oral cavity and partially reduced to nitrite by the oral microflora [30]. In addition to nitrate or nitrite under conditions that result in endogenous nitrosation being classified as "probably carcinogenic to humans", nitrite is also important in biochemistry as a source of nitric oxide, a potent vasodilator [31]. Nitrate and nitrite, through ultraviolet light exposure to skin, mammalian nitrate/nitrite reductases in tissues, and nitric oxide synthase enzymes, are converted to a diverse group of metabolites including nitric oxide, a powerful vasodilator, and potentially influence the risk of developing cardiovascular diseases [32]. Tripp et al. found that in sialoadenectomized rats, inhibition of nitric oxide synthase activity was exacerbated, implying that factors from the salivary glands influence gastric nitric oxide formation [33]. Björne et al. reported that nitrite-containing human saliva given luminally increases gastric mucosal blood flow, and these nitrite-mediated effects were associated with the generation of nitric oxide [34]. Webb et al. reported that blood pressure-lowering, vasoprotective, and antiplatelet properties can even be achieved by dietary nitrates, and interruption of the enterosalivary conversion of nitrate to nitrite (facilitated by bacterial anaerobes situated on the surface of the tongue) prevented the rise in plasma nitrite, blocked the decrease in blood pressure, and abolished the inhibitory effects on platelet aggregation [18]. Those authors stated that the vasoprotective effects were attributable to the activity of nitrite converted from ingested nitrate. Because the salivary glands are very important in nitrate transport and nitric oxide homeostasis, the sialoadenectomy procedure might affect the enterosalivary conversion pathway and ultimately lead to an increased risk of CHD [17].

Nevertheless, one should be reminded that this is an observational study, and the findings in this study must be further investigated before a true hypothesis is made.

Also, like much health insurance database analytical research, it comes with the possibility of surveillance bias. Patients receiving a sialoadenectomy are more likely to visit outpatient clinics for follow-up, and this might lead to an increased detection of CHD due to the increased exposure to medical services. Furthermore, another important limitation is that the LHID2005 provides no information on body mass index (BMI). Nevertheless, in order to eliminate the potential effects of BMI on the association between sialoadenectomy and CHD, we have used obesity in place of BMI in this study.

Although this was an observational study, the result that a sialoadenectomy might be associated with an increased risk of developing CHD is worth further investigation. Clinically, it is suggested that physicians might take the findings in this study into considerations in determining the risks and benefits of the sialoadenectomy procedure and even consider different approaches such as a partial sialoadenectomy according to the patient’s gender and other comorbidities [35].
