# Correction: A sialoadenectomy is associated with an increased risk of coronary heart disease: A three-year follow-up study

**DOI:** 10.1371/journal.pone.0243568

**Published:** 2021-01-08

**Authors:** Shih-Han Hung, Chin-Hui Su, Herng-Ching Lin, Chung-Chien Huang, Senyeong Kao

The affiliation for the 2^nd^ author is incorrect. Chin-Hui Su is affiliated with both #2 and #3. Affiliation #2 is: Department of Otolaryngology, College of Medicine, School of Medicine, Taipei Medical University, Taipei, Taiwan
